# A bench experiment to investigate the time available to replace a near‐depleted E‐size nitrous oxide cylinder when using a ‘mobile manifold’ supply system

**DOI:** 10.1111/anae.70202

**Published:** 2026-03-10

**Authors:** Scarlett Tankard, Cliff Shelton

**Affiliations:** ^1^ Wythenshawe Hospital Manchester UK

Nitrous oxide is a long‐lived greenhouse gas with a global warming potential approximately 300 times that of carbon dioxide. Historically, it had an important role in anaesthetic practice. However, as low‐solubility volatile anaesthetic agents and total intravenous anaesthesia have become more common, its clinical use has waned [1]. Piped supply of nitrous oxide is associated with a high proportion of waste, with as much as 99% being wasted before patient use [2]. In 2024, a national position statement was released, advocating a move away from piped nitrous oxide towards cylinder supply in institutions where the gas is still used [1].

The conventional way to supply nitrous oxide by cylinder is to attach it to the anaesthetic machine via a yoke [3]. However, many modern machines do not have this facility, and some institutions have therefore adopted a ‘mobile manifold’ approach. This is where a small nitrous oxide cylinder (e.g. D‐ or E‐size) is attached to a 4‐bar pressure regulator with a Schrader socket, allowing attachment via the flexible hose and Schrader probe (in the same way that portable oxygen cylinders are attached to transport ventilators). This means that the nitrous oxide supply is registered by the anaesthetic machine as a 4‐bar pipeline supply, rather than a cylinder supply (which has a nominal pressure of 44 bar when full, that is, the saturated vapour pressure of nitrous oxide) [3].

Nitrous oxide is supplied as a liquefied gas, meaning that the cylinder pressure will only start to fall once all the liquid gas has been vaporised. As such, the pressure gauge only falls once most of the content (approximately 89%) has been depleted. This can make it challenging to determine how much nitrous oxide remains in a cylinder during use [3]. As we have moved towards point‐of‐care nitrous oxide supply, some colleagues have raised concerns regarding the implications of the cylinder running low midway through a case. These include whether they need to swap the cylinder once the cylinder gauge pressure starts to fall and how much time they have to swap the cylinder once the gas supplied via the regulator starts to fall below nominal pipeline pressure and the low pipeline pressure alarm sounds. We designed a bench experiment to characterise the gas supply during the complete depletion of an E‐size nitrous oxide cylinder connected to an anaesthetic machine via a mobile manifold.

**Figure 1:**
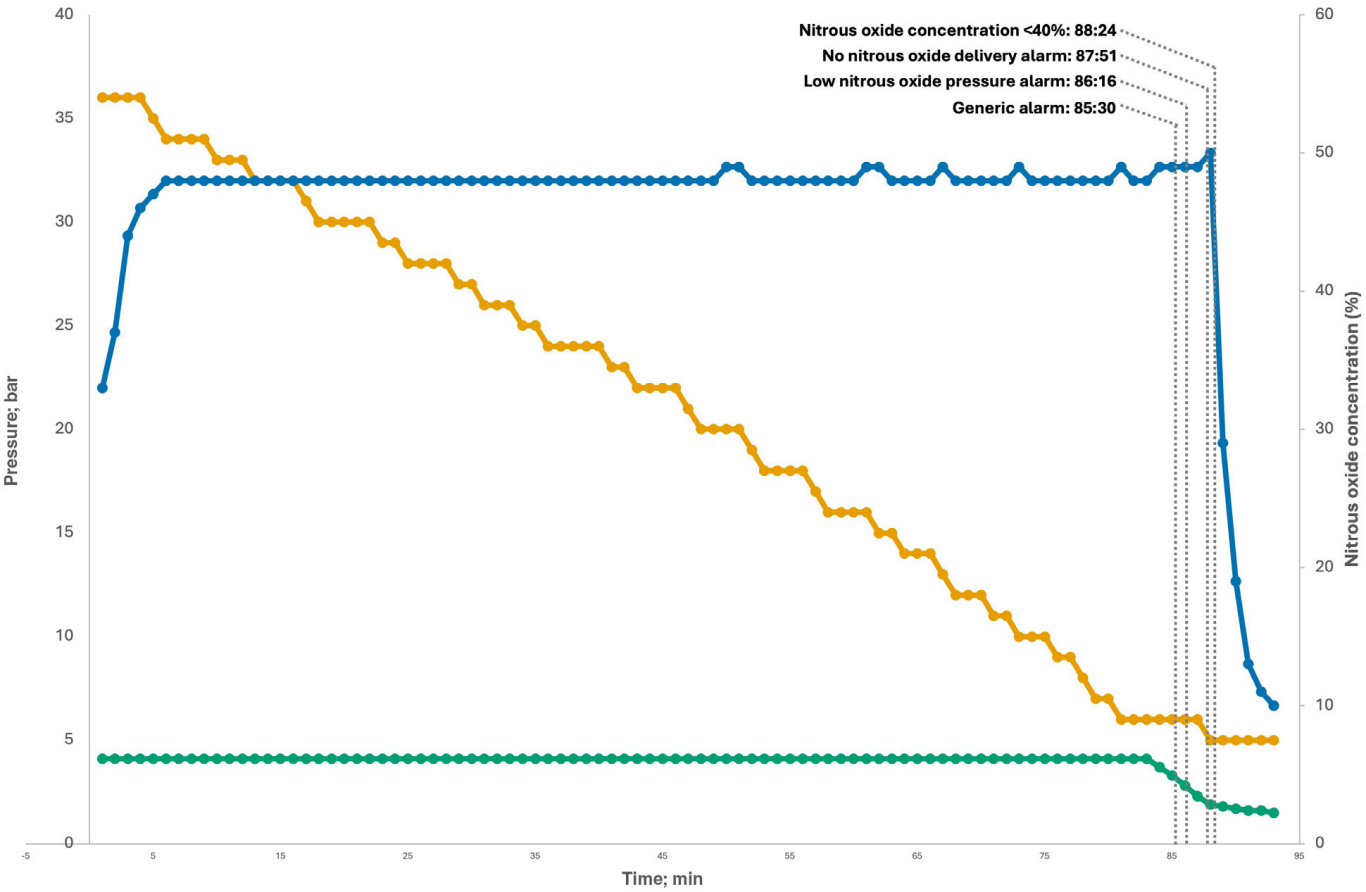
Cylinder pressure (yellow) and pipeline pressure (green), and measured nitrous oxide concentration (blue), during the depletion of a near‐empty E‐size nitrous oxide cylinder attached to an anaesthetic machine via a mobile manifold. Alarm timings are marked by dotted lines.

Our experimental method involved connecting a near‐empty E‐size nitrous oxide cylinder (which had been discarded from clinical use and was awaiting return to the gas supply company) to an anaesthetic machine (Primus, Dräger, Lübeck, Germany), via a regulator (Medireg II, GCE Group, Steinhausen, Switzerland). Oxygen was supplied to the anaesthetic machine via the hospital pipeline system. We attached a 2 l reservoir bag to the end of a circle breathing system (Flexitube 1.6 m, Intersurgical Ltd, Wokingham, UK). We set the gas mix to 50% oxygen and 50% nitrous oxide, the fresh gas flow to 5 l.min^‐1^ and set the ventilator to deliver 500 ml tidal volumes at a rate of 12 min^‐1^. We observed the anaesthetic machine and the nitrous oxide regulator continuously until the nitrous oxide concentration (read from the integrated sidestream gas analyser, which samples from the y‐connector of the circle circuit) fell to 10%. We documented the nitrous oxide gauge pressure (read from the cylinder gauge), the nitrous oxide pipeline pressure (read from the anaesthetic machine) and the nitrous oxide concentration (read from the anaesthetic machine) at 1‐min intervals. We also documented the times at which gas pressure and delivery alarms sounded and the time when the concentration of nitrous oxide fell to <40% (Fig. 1).

This bench experiment shows that even at high fresh gas flows and concentrations (i.e. 5 l.min^‐1^ and 50% nitrous oxide), an E‐size nitrous oxide cylinder with a cylinder gauge pressure of 36 bar (indicating approximately 91% depletion of the original contents) will continue to supply nitrous oxide at nominal pipeline pressure for over 80 min. In this experiment, pipeline pressure did not fall until the pressure in the cylinder dropped to 6 bar (indicating over 98% depletion). The time between the first anaesthetic machine alarm and the concentration of nitrous oxide falling to <40% was 2 min 54 s.

These data should help colleagues who use mobile manifold systems for nitrous oxide delivery to consider the optimal timing for cylinder changes and provide reassurance that there is adequate time to swap a nitrous oxide cylinder or switch to alternative hypnotic drugs, even in the event of the cylinder becoming so depleted that the low gas pressure alarm sounds. This is relevant because it has been noted by some institutions that point‐of‐care cylinders can be inefficient if swapped for full cylinders too early and colleagues have stated the need for education on when to exchange cylinders [4]. It should be said that the times documented above are likely to be extended proportionally when low‐flow anaesthesia is used.

A limitation of this experiment is that it only examines the performance of a single model of anaesthetic machine and a single model of regulator, in the context of a relatively high fresh gas flow rate. Future work could seek to replicate these findings with other equipment. In view of the significant environmental impacts of nitrous oxide, we would recommend that any such work be done with near‐depleted cylinders which are scheduled for return to the supplier (as done here) to avoid contributing to the problem of nitrous oxide waste.
